# Role of Attention Bias as a Moderator and Mediator in the Bi‐Directional Association Between Body Dysmorphic Concerns and Psychotic Experiences Among Adolescents

**DOI:** 10.1111/eip.70109

**Published:** 2025-11-16

**Authors:** Feten Fekih‐Romdhane, Sarah El Hayek, Georges Haddad, Souheil Hallit

**Affiliations:** ^1^ The Tunisian Center of Early Intervention in Psychosis, Department of Psychiatry “Ibn Omrane”, Razi Hospital Manouba Tunisia; ^2^ Faculty of Medicine of Tunis Tunis El Manar University Tunis Tunisia; ^3^ School of Medicine and Medical Sciences Holy Spirit University of Kaslik Jounieh Lebanon; ^4^ Psychiatry Department Psychiatric Hospital of the Cross Jal Eddib Lebanon; ^5^ Applied Science Research Center Applied Science Private University Amman Jordan

**Keywords:** adolescence, attention bias, body dysmorphic disorder, cognitive bias, psychotic experiences

## Abstract

**Background:**

The link between body dysmorphic disorder (BDD) and psychosis remains under‐researched and the mechanisms behind it are still unknown and poorly understood. This study aimed to test the hypothesis that attentional bias moderates and mediates the positive bi‐directional association between BDD and psychotic experiences (PEs).

**Methods:**

The study was cross‐sectional. It took part between December 2023 and January 2024 among 336 adolescents.

**Results:**

Attention bias partially mediated the association between PEs and BDD symptoms (indirect effect: Beta = 0.07; Boot SE = 0.02; Boot CI 0.03; 0.13), as well as between BDD symptoms and PEs (indirect effect: Beta = 0.05; Boot SE = 0.02; Boot CI 0.02; 0.09). Moreover, attention bias moderated the association between PEs and BDD symptoms (Beta = 0.02; *p* = 0.006). At moderate (Beta = 0.31; *p* < 0.001) and high (Beta = 0.47; *p* < 0.001) levels of attention bias, higher PEs were associated with greater BDD symptoms.

**Conclusion:**

Findings preliminarily suggest that the relationship linking these conditions seems to be complex and bidirectional.

## 1 Introduction

Throughout history, body dysmorphic disorder (BDD) has been a concept challenging to describe and classify in psychiatric epistemology, varying from symptoms to syndrome, and from psychosis to neurosis (Recio‐Barbero et al. 2019). There remain doubts about its psychopathological nature, and whether it manifests as an overestimated idea, a delirious idea or an obsessive idea (Recio‐Barbero et al. 2019). BDD is commonly defined as excessive preoccupation with defect(s) or perceived flaw(s) in physical appearance that is either slight or not noticeable by others, along with mental acts and/or repetitive behaviours occurring in response to the preoccupation. The concept of BDD has evolved in the different editions of the Diagnostic and Statistical Manual of Mental Disorders (DSM). In the Fifth Edition (DSM‐5), BDD is classified under the category of ‘obsessive–compulsive disorder and related disorders’, pointing out the importance of acts and repetitive behaviours related to the anxiety about physical defects, whereas the delirious presentation is included as a specifier of the presence or absence of ‘insight’ rather than as a delusional disorder of a somatic nature (as was the case in older versions) (American Psychiatric Association 2013). Following this conceptualization, the permanent preoccupation with one's body imperfections can be regarded as an obsession, while searching for reaffirmation, camouflage behaviour and repetitive checking may be seen as forms of compulsive behaviours. Historically, BDD was considered by several authors as a variant or prodrome of schizophrenia (Zaidens 1950), and was even contemplated as the only presenting manifestation in the prodromal phase of disease (Sadock et al. 2000). Despite this, BDD has been relatively neglected in empirical research, particularly regarding its overlap with psychotic symptoms. Renewed interest has only recently emerged, with case reports suggesting that BDD can occur at early or prodromal stages of schizophrenia (Abbassi et al. 2018; Durak et al. 2019; Lucchelli et al. 2006). Yet, systematic empirical studies remain scarce. This underscores the need to more rigorously examine the nature of the link between BDD and psychotic experiences (PEs), especially in adolescent, non‐clinical populations.

### 1.1 The Relationship Between BDD and Psychotic Symptoms

A large body of evidence from clinical studies pointed to the coexistence of BDD and psychotic symptoms. Research focusing on the co‐occurrence of both entities emerged from clinical samples of either patients with psychotic disorders or those with BDD. Studies performed in patients diagnosed with schizophrenia have consistently indicated an increased proneness to disturbed sensations in the way these patients experience their own bodies, which subsequently impacts the severity and persistence of their psychotic symptoms (Marshall et al. 2020; Waite et al. 2023, 2019). More recently, abnormal bodily phenomena have also been observed at the earlier stages of the disease, such as in patients with First Episode Psychosis (Röhricht et al. 2020; Stanghellini et al. 2012) or those at ultra‐high risk (Madeira et al. 2016), and were suggested as potential early markers of psychosis and relevant determinants of the course and prognosis of psychotic diseases. A comparative study showed that patients with BDD endorsed not only appearance‐related, but also control and thought alienation delusional ideation to the same extent as those diagnosed with schizophrenia (Rossell et al. 2020). In addition, the BDD group was distressed by persecutory and referential delusions to a comparable degree with the schizophrenia group, suggesting an overlap in key symptom dimensions of the two disorders (Rossell et al. 2020). Owing to the similar clinical presentations and high co‐occurrence between BDD and psychosis, a complex and bidirectional relationship can be hypothesised. Such speculation is important to explore as it may serve as a basis to understand the pathology of both diseases, and could inform early detection and prevention strategies. A bidirectional association may foster a vicious cycle whereby BDD can lead to more severe PEs and PEs may exacerbate BDD.

Potential factors and mechanisms that might explain the pathways between BDD and psychosis are unclear and not yet elucidated. Malcolm et al. (Malcolm et al. 2022) suggested that the way patients perceive their own appearance can be regarded as a source of threat that ‘may both fuel and be exacerbated by paranoia’. The authors attempted to explain this relationship by proposing that negative feelings about one's own body may result in feelings of inferiority and a negative self‐image, which leads in turn to increased vulnerability and the occurrence of persecutory delusions (Malcolm et al. 2022). It is of note, however, that the study was performed in a relatively small sample consisting of females only, and body image concerns were restricted to (‘feeling larger’). In non‐clinical samples, body image concerns were shown to be linked to elevated levels of paranoid thoughts through the indirect effect of social criticism (Bagrowska et al. 2022). Another potential explaining mechanism of the relationship BDD–psychosis that will be the subject of this paper is attention bias.

### 1.2 Attention Bias as a Mediator and Moderator in the Bidirectional Association Between BDD and PEs

This study proposes to test a hypothetical model to test the bi‐directionality of the association between BDD symptoms and PEs (i.e., delusions and hallucinations that are experienced by individuals from the general population and manifest at a lesser intensity or duration than clinical‐level psychotic symptoms) (Fusar‐Poli et al. 2017), and to explore the possible mediating and moderating roles of attention bias in these associations. The present conceptual framework follows Young's (2015) position, which advocates for circular causality in psychiatry, and proposes a non‐linear, hybrid model of connections between symptoms and mental illness. Attention bias refers to ‘the tendency to prioritise the processing of certain types of stimuli over others’ (Azriel and Bar‐Haim 2020). It is largely recognised that selective attention bias accounts for biassed attention towards threat or disorder‐related stimuli (MacLeod et al. 2002).

The cognitive theory of BDD posits that attention bias towards perceived flaws in appearance is implicated in its aetiology, exacerbation and persistence (for systematic review and meta‐analysis, see Johnson et al. 2018). Findings from clinical and neurocognitive research demonstrated that people with BDD selectively attend to minor appearance imperfections or specific aspects of their appearance, suggesting a bias for detailed rather than global visual processing (Feusner, Moody, et al. 2010; Feusner, Moller, et al. 2010), and pointing to an overuse of detail‐oriented left hemisphere at exposure to pictures of their own faces relative to healthy controls (Feusner, Moody, et al. 2010). Subsequently, selective attention biases towards perceived threats (flaws in physical appearance in the case of BDD), are suggested to trigger negative feelings, which, in turn, result in an array of time‐consuming behaviours to regulate emotions (including incessant mirror checking or seeking out cosmetic procedures) (Johnson et al. 2018). Johnson et al. (2020) investigated Australian students using the dot‐probe task and revealed that attention bias towards target word stimuli (e.g., attractive, pretty, chiselled) predicted dysmorphic concern in males and females. Similar findings were also obtained from several other previous studies (Fang and Wilhelm 2015; Jin et al. 2018; Johnson et al. 2018). Another study showed that, compared to individuals with social phobia and controls, those with BDD were the only ones to display biassed (heightened selective) visual attention to the defect imagined when viewing unfamiliar as well as their own faces (Grocholewski et al. 2012). The impact of biassed attention on BDD was suggested to set up a negative feedback loop in which BDD symptoms are, in turn, perpetuated by where visual attention is directed (Moody et al. 2017).

At the same time, there is enough evidence to support that cognitive biases are relevant to psychotic symptoms (e.g., Gawęda et al. 2015; McLean et al. 2017; Randjbar et al. 2011; Ross et al. 2015; So et al. 2015). In particular, attention to threat bias was consistently shown to significantly correlate with PEs in healthy individuals from the community, and to play, therefore, a major role in the early phases of psychotic symptoms (Prochwicz and Kłosowska 2017; Prochwicz et al. 2017). A range of studies has investigated the role of attention bias in the development of PEs, and highlighted similar findings (Aakre et al. 2009; Arguedas et al. 2006; Bentall et al. 2001; Green et al. 2001; Kinderman et al. 2003; Phillips et al. 2000). In their literature review of empirical knowledge from studies comparing individuals with and without a need for care, Underwood et al. (2016) conclude that attentional bias towards threat plays a determinant role in the onset, exacerbation and maintenance of psychotic symptoms, and that these biases should be regarded ‘not as peripheral, but etiologically relevant’. All these findings and observations have provided additional support for the cognitive theory of positive psychotic symptoms (Garety et al. 2001), and have driven increasing attention towards the design and implementation of psychological interventions directly targeting attentional biases for people with psychosis (e.g., Garety et al. 2015; Moritz et al. 2011).

**FIGURE 1:**
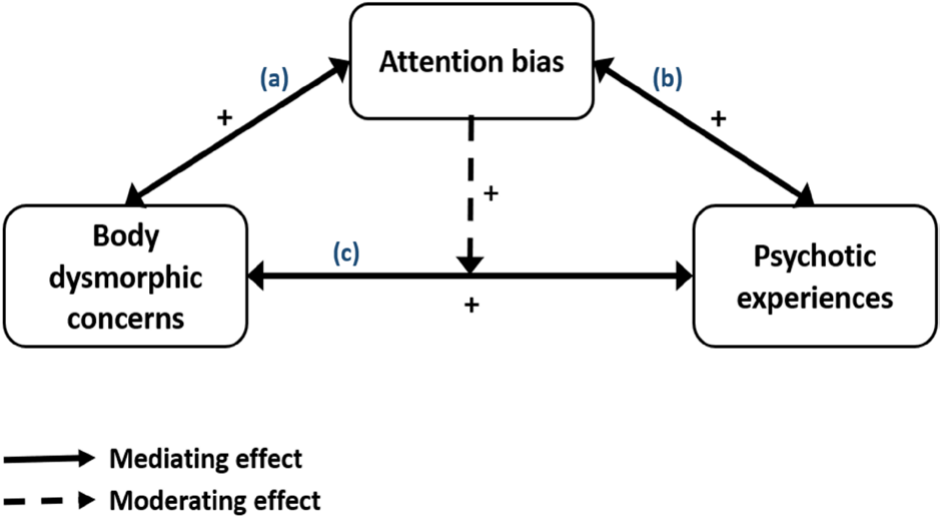
Conceptual framework.

A mediator variable is conceptualised as the explanatory process that underlies a relationship between an independent variable and a dependent variable. The investigation of mediation effects allows for determining whether a third factor helps explain or accounts for the relationship between these variables (Rose et al. 2004). To date, the role that attention bias might play in the relationship between BDD and PEs has not yet been directly studied. In this study, we hypothesise that biassed attention might be a cognitive mechanism by which the association between BDD and PEs can be explained, at least in part. In other words, it is theoretically assumed that BDD may have both a direct (path c in Figure 1) and an indirect effect (paths a + b in Figure 1) on PEs through attention bias, and vice versa. Interestingly, the same factor can act as either a moderator, a mediator or both (Rose et al. 2004). Therefore, in addition to potentially acting as a mediator in the relationship between BDD and PEs, it is reasonable to postulate that attention bias can also serve as a trigger or an aggravating factor for this relationship, increasing the likelihood that someone with BDD would develop PEs. By contrast to a mediator, a moderating variable is a factor that influences the direction or the strength of a relationship between a criterion variable and a predictor variable (Rose et al. 2004). The reason why attention bias could serve as also a moderator in this relationship is that, given the evidence presented above, it can be reasoned that BDD experiences are more likely to have adverse effects on PEs when the person has high levels of attention bias. Based on previous literature, attention bias can be presumed as a contextual variable that renders the effect of BDD on PEs significant and more robust. In other terms, a significant association between BDD and PEs may depend on, or emerge only in the presence of biassed attention. Thus, the investigation of the moderating effect enables determining whether there exist certain conditions under which BDD predicts PEs. Specifically, we hypothesise in the present study that attention bias may serve as a moderator such that those who experience BDD but who also have high levels of attention bias will be more likely to experience PEs than those with similar experiences of BDD but lower levels of attention bias. Our approach of examining, for the first time, both the mediating and moderating roles of attention bias in this relationship would not only allow for more accurate conclusions, but would potentially have major implications for prevention and intervention (Rose et al. 2004).

### 1.3 Rationale

There are several reasons why this study was undertaken. First, the link between BDD and psychosis remains under‐researched and the mechanisms behind it are still unknown and poorly understood. Examining potentially preventable mediating/moderating factors can pave the way towards the identification of targets for future prevention efforts. Second, we could not find any previous studies investigating the association between BDD and PEs within clinical or non‐clinical contexts in Arab countries. Prior research has shown that BDD (Dixon and Marques 2017) and PEs (Fekih‐Romdhane, Obeid, et al. 2023) are culturally dependent. Besides, the recognition of emotional facial expressions is not culturally universal (Crivelli et al. 2016; Gendron et al. 2014), and humans from various cultural backgrounds attend to negative or positive information differently (Grossmann et al. 2012). However, most of the existing literature and current knowledge on attentional bias to negative stimuli stem from Western countries and populations, who cannot be considered representative of the broader global population. Therefore, this research is a unique attempt to bridge this gap by investigating the topic in a non‐Western, under‐researched context. Third, investigating the complex relationship between attention bias, BDD and PEs in a non‐clinical population could inform theoretical models on the aetiology of the two conditions, which is still largely unknown. Fourth, adolescence is a critical period of great changes to the human body, that significantly affect one's perceived physical appearance (Roberts et al. 2024), and potentially increase negative cognitive biases (Slavny et al. 2019). In addition, adolescence is a peak age of onset of different mental disorders, including BDD and psychotic disorders (Solmi et al. 2022). Therefore, investigating this topic during adolescence can be of high relevance. Altogether, this research has as a main goal to test the hypothesis that attentional bias moderates and mediates the positive bi‐directional association between BDD and PEs, using a cross‐sectional design and a population‐based adolescent sample.

## 2 Method

### 2.1 Design, Sample and Procedure

The study has a cross‐sectional design and took part between December 2023 and January 2024. A survey was developed on Google Forms using the snowball sampling method. The survey had a completion time of around 15 min. It was distributed to potential participants via social media networks and messaging applications (Messenger, Instagram and WhatsApp) by contacting Lebanese high schools. The inclusion criteria were the following: (1) being aged 15–18 years (2) having access to the Internet, (3) being a Lebanese resident and citizen and (4) willing to participate voluntarily in the research. We excluded participants who declined to complete the survey. The study protocol was approved by the ethics committee of Notre Dame des secours university hospital. Participation was on a voluntary basis. Confidentiality and anonymity were ensured. Before taking part in the study, each participant was informed of their right to withdraw from the study at any point before submission, and was asked to provide written informed consent. For any participant under the age of 16 years, informed consent to participate was obtained from the parents or legal guardians. No compensation was offered for participation. Finally, participants were informed about the potential emotional discomfort that the questionnaire may induce and were provided with details on mental health resources.

### 2.2 Minimal Sample Size Calculation

Using the G power software where $f^{2}$ = 5.2%, power *β* = 80%, an *α* error of 5%, and nine factors as variables to be entered in the model, a minimal sample of 306 was deemed necessary.

### 2.3 Survey

The first section of the survey on sociodemographic information included age, sex, residential location, educational attainment and the Household Crowding Index (HCI). The HCI is determined by dividing the total number of individuals residing in a household by the number of rooms (excluding the kitchen) in that household. The second section of the survey contained the following three measurement instruments:

#### 2.3.1 The Dysmorphic Concern Questionnaire)

The Dysmorphic Concern Questionnaire (DCQ) is a self‐administered tool composed of seven items (e.g., ‘Have you ever been very concerned about some aspect of your physical appearance’ or ‘Have you ever considered yourself misformed or misshapen in some way [e.g. nose/hair/skin/sexual organs/overall body build]’) (Oosthuizen et al. 1998). Each item is rated on a 4‐point scale ranging from 0 (Not at all) to 3 (Much more than most people) (score range between 0 and 21). Higher scores reflect greater BDD symptoms. Scores ≥ 9 have been suggested to indicate high risk of BDD from community samples. The version of the DCQ validated in Arabic was adopted (Fekih‐Romdhane et al. 2024). Cronbach's alpha was 0.88 in the present sample.

#### 2.3.2 The Psychotic‐Like Experiences Questionnaire for Children

This is a self‐administered scale composed of nine that assess adolescents' endorsement of different PEs (e.g., ‘Have you ever thought that you were being followed or spied upon?’ or ‘Have you ever heard voices that other people could not hear?’) (Laurens et al. 2012, 2007). Items are rated on a 3‐point scale as follows: 0 (not true); 1 (somewhat true) and 2 (certainly true) (score range between 0 and 18). The version validated in Arabic of the Psychotic‐Like Experiences Questionnaire for Children (PLEQ‐C) was utilised, which exhibited good psychometric properties (*α* = 0.85) (Fekih‐Romdhane, Malaeb, et al. 2023). In our study, Cronbach alpha was 0.81.

#### 2.3.3 The Attention Bias Questionnaire

This scale is composed of nine items assessing respondents' experience of attention bias towards threats (e.g., ‘It is difficult for me not to look at threatening things’, ‘I am vigilant and alert towards threats in the surroundings’) (Azriel et al. 2022). Respondents were instructed to refer to the general term ‘threat’ as any specific thing(s) (objects, people, animals, situations) that threaten them personally or that are stressful for them. Each item is scored on a 5‐point Likert‐type scale ranging from 0 (‘not at all’) to 4 (‘to a great extent’) (score range between 0 and 36). Before its use in this study, the Attention Bias Questionnaire (ABQ) was translated to Arabic language following international norms and recommendations and using the forward‐backward translation method (Van Widenfelt et al. 2005). As an initial step, a translator from Lebanon who was unrelated to the research conducted the translation from the English to the Arabic language. Then, a Lebanese psychologist who has full working proficiency in English back‐translated the version in Arabic obtained to English. Any specific and/or literal translation was balanced by the translation team. Afterwards, the initial and translated English versions were compared by a committee of experts composed of the two translators and the research team with the aim of identifying any inconsistencies and ensuring the accuracy of the translation (Fenn et al. 2020). To detect any misunderstanding of the items' wording and determine the ease of items' interpretation, an adaptation of the scale to the Arab context was done (Ambuehl and Inauen 2022). Last, a pilot study was performed on 20 participants to guarantee that all questions were correctly understood; no changes were applied after the pilot study.

### 2.4 Statistical Analysis

The SPSS software v.25 was used for the statistical analysis. The skewness and kurtosis values varied between −1.96 and +1.96, indicating that the BDQ and PLEQ‐C scores were considered normally distributed (George 2011). The Pearson test was used to correlate two continuous variables and the Student‐*t* was used to compare two means. To calculate three pathways, we used the PROCESS SPSS Macro version 3.4, model four (Hayes 2017), taking dysmorphic concerns as an independent variable, attention bias as the moderator and PEs as the dependent variable. Pathway A examined the association between PEs and attention bias; Pathway B determined the effect of attention bias on body dysmorphic concerns, and Pathways C and C′ estimated the total and direct effects of PEs on body dysmorphic concerns. If the Bootstrapped confidence interval did not pass by zero, significance was deemed present (Hayes 2017). Results were adjusted over all variables that showed a *p* < 0.25 in the bivariate analysis. *p* < 0.05 was deemed statistically significant.

## 3 Results

Three hundred thirty‐six adolescents participated in this study, with a mean age of 15.69 ± 1.10 years and 63.4% females. Other descriptive statistics of the sample can be found in Table 1. Moreover, 68 (20.2%) adolescents had body dysmorphic concerns (DCQ scores ≥ 9).

**TABLE 1:** Sociodemographic and other characteristics of the sample (*N* = 336).

Variable	*N* (%)
Sex	
Female	213 (63.4%)
Male	123 (36.6%)

	Mean ± SD
Age (years)	15.69 ± 1.10
HCI	1.13 ± 0.63
Body dysmorphic concerns	4.60 ± 4.85
Psychotic experiences	5.12 ± 3.96
Attention bias	14.10 ± 8.07

Abbreviation: HCI, household crowding index (persons/room).

### 3.1 Bivariate Analysis of Factors Linked to Body Dysmorphic Concerns and PEs

A significantly higher mean BDC score was found in females compared to males (5.09 ± 4.91 vs. 3.76 ± 4.64, *t*_(334)_ = −2.46, *p* = 0.015). Moreover, older age (*r* = 0.11) and higher attention bias (*r* = 0.27) were significantly associated with more body dysmorphic concerns (Table 3).

There was no significant difference between males and females in terms of PEs (5.61 ± 4.53 vs. 4.83 ± 3.57; *t*_(334)_ = 1.74; *p* = 0.082). Furthermore, older age (*r* = 0.17) and higher attention bias (*r* = 0.30) were significantly related to more body dysmorphic concerns (Table 2).

**TABLE 2:** Correlations of body dysmorphic concerns with continuous variables.

	1	2	3	4
1. Body dysmorphic concerns	1			
2. Psychotic experiences	0.30***	1		
3. Age	0.11*	0.17**	1	
4. HCI	0.06	0.06	0.11*	1
5. Attention bias	0.27***	0.30***	0.06	−0.03

Abbreviation: HCI, household crowding index (persons/room).

* *p* < 0.05.

** *p* < 0.01.

*** *p* < 0.001.

### 3.2 Mediation Analysis of Attention Bias Between PEs and Body Dysmorphic Concerns

**FIGURE 2:**
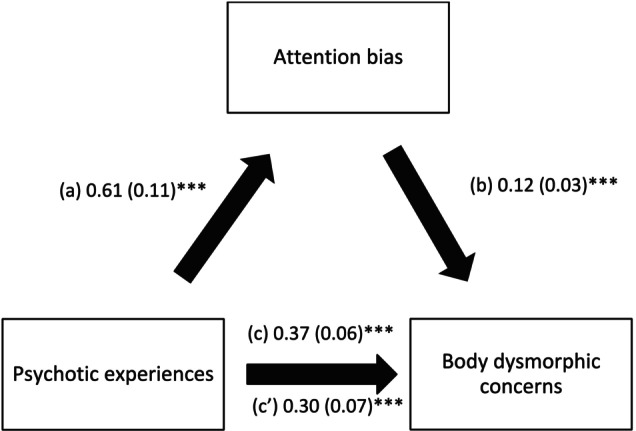
Mediating effect of Attention bias between psychotic experiences and body dysmorphic concerns. (a) Relation between psychotic experiences and attention bias (*R* ^2^ = 0.092); (b) Relation between attention bias and body dysmorphic concerns (*R* ^2^ = 0.155); (c) Total effect of psychotic experiences on body dysmorphic concerns (*R* ^2^ = 0.119); (c′) Direct effect of psychotic experiences on body dysmorphic concerns. Numbers are displayed as regression coefficients (standard error). ***p < 0.001.

The results of the mediation analysis were adjusted over sex and age. Attention bias partially mediated the relationship between PEs and body dysmorphic concerns (indirect effect: Beta = 0.07; Boot SE = 0.02; Boot CI 0.03; 0.13). Higher PEs were significantly associated with more attention bias, whereas more attention bias was significantly related to more body dysmorphic concerns. Finally, higher PEs were significantly linked to more body dysmorphic concerns (Figure 2).

### 3.3 Mediation Analysis of Attention Bias Between Body Dysmorphic Concerns and PEs

**FIGURE 3:**
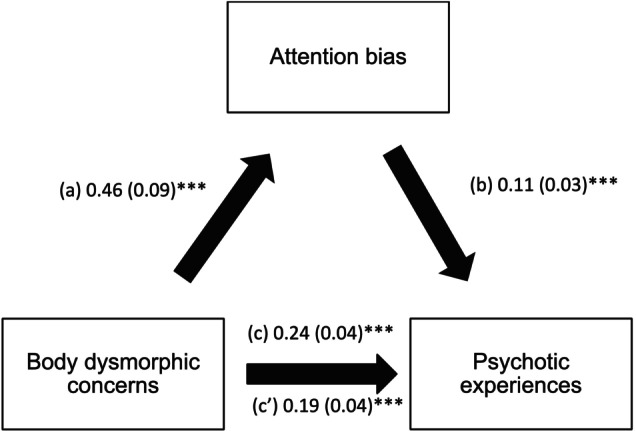
Mediating effect of Attention bias between body dysmorphic concerns and psychotic experiences. (a) Relation between body dysmorphic concerns and attention bias (*R* ^2^ = 0.079); (b) Relation between attention bias and psychotic experiences (*R* ^2^ = 0.170); (c) Total effect of body dysmorphic concerns on psychotic experiences (*R* ^2^ = 0.123); (c′) Direct effect of body dysmorphic concerns on psychotic experiences. Numbers are displayed as regression coefficients (standard error). ***p < 0.001.

The results of the mediation analysis were adjusted over sex and age. Attention bias partially mediated the association between body dysmorphic concerns and PEs (indirect effect: Beta = 0.05; Boot SE = 0.02; Boot CI 0.02; 0.09). Higher body dysmorphic concerns were significantly linked to more attention bias, whereas more attention bias was significantly linked to more PEs. Finally, higher body dysmorphic concerns were significantly linked to more PEs (Figure 3).

### 3.4 Moderation Analysis of Attention Bias Between PEs and Body Dysmorphic Concerns

**FIGURE 4:**
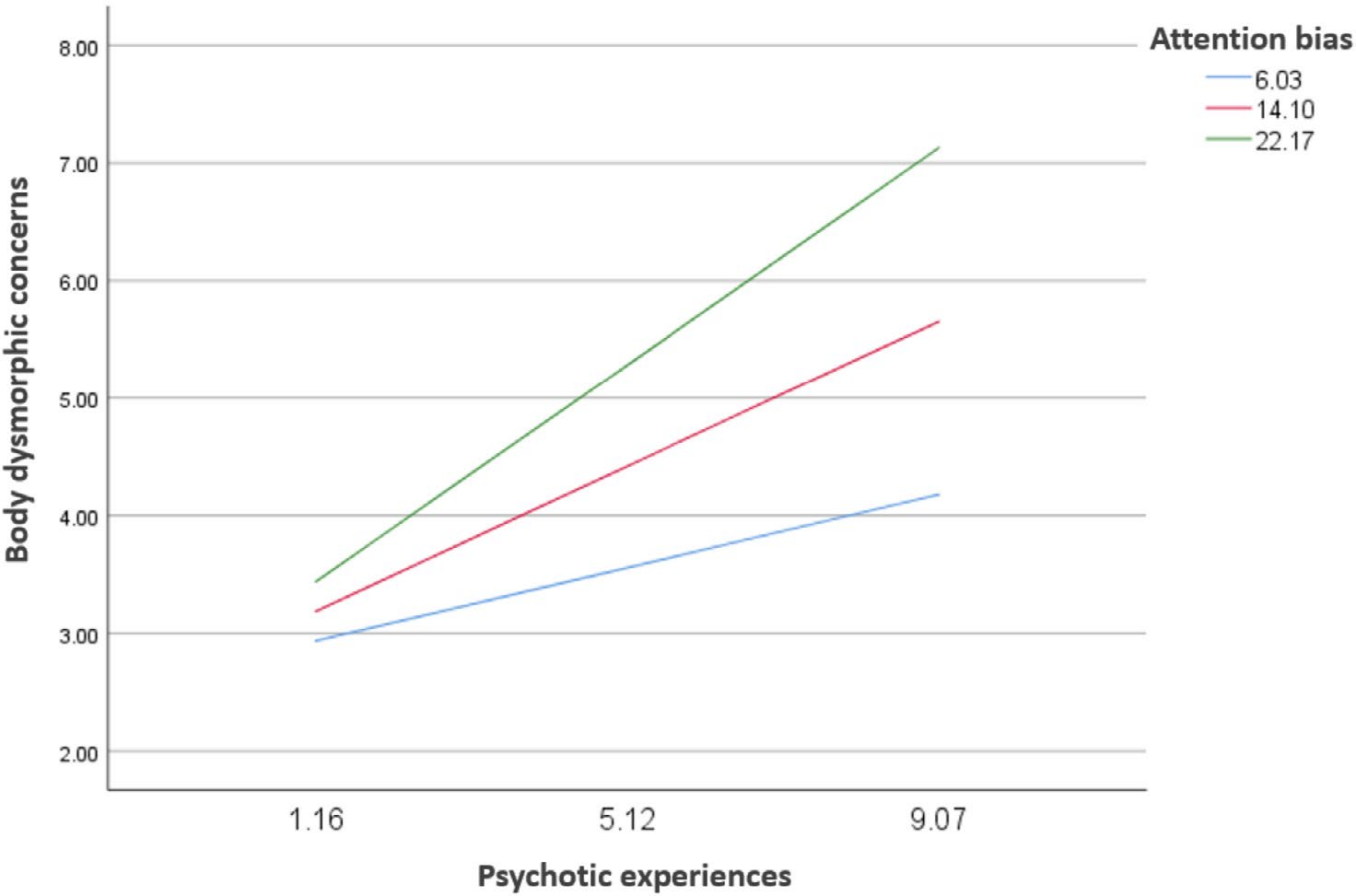
Moderation analysis of attention bias between psychotic experiences and body dysmorphic concerns.

Attention bias moderated the association between PEs and body dysmorphic concerns (Beta = 0.02; *p* = 0.006) (Table 3; Figure 4). At moderate (Beta = 0.31; *p* < 0.001) and high (Beta = 0.47; *p* < 0.001) levels of attention bias, higher PEs were significantly associated with more body dysmorphic concerns (Table 4).

**TABLE 3:** Moderation analysis taking psychotic experiences as the independent variable, attention bias as the moderator and body dysmorphic concerns as the dependent variable.

	Beta	*t*	*p*	95% CI
Psychotic experiences	0.04	0.37	0.712	−0.18; 0.26
Attention bias	0.01	0.18	0.860	−0.09; 0.11
Interaction psychotic experiences by attention bias	0.02	2.78	0.006	0.01; 0.03^a^

^a^ Indicates significant moderation; results adjusted over age and sex.

**TABLE 4:** Conditional effects of the focal predictor (psychotic experiences) at values of the moderator.

Attention bias	Beta	*t*	*p*	95% CI
Low (= 6.03)	0.16	1.91	0.057	−0.01; 0.32
Moderate (= 14.10)	0.31	4.75	**< 0.001**	0.18; 0.44
High (= 22.17)	0.47	5.20	**< 0.001**	0.29; 0.64

*Note:* Numbers in bold indicate significant *p* values.

### 3.5 Moderation Analysis of Attention Bias Between Body Dysmorphic Concerns and PEs

Attention bias did not moderate the association between body dysmorphic concerns and PEs (Beta = 0.01; *t* = 1.94; *p* = 0.053; 95% CI −0.001; 0.02).

## 4 Discussion

Both BDD and early psychosis are complex conditions that can be difficult to treat (Fang and Wilhelm 2015; Haddad and Chaudhry 2012), and this difficulty becomes even more pronounced when the two conditions co‐exist (Röhricht et al. 2020). Besides, the concomitant occurrence of body dysmorphic concerns and psychosis seems to lead to lower levels of quality of life, psychological well‐being and overall health (Waite et al. 2023, 2019). Therefore, further investigation of this under‐researched topic is required to determine potential factors that may explain the bidirectional relationship between BDD and psychosis along the continuum of both conditions' severity in the general population, and can thus be targeted in interventions. The bidirectional hypothesis was explored in a cross‐sectional population‐based study, while applying adjustments for important confounding variables. The study's hypothesis was supported. Findings showed that attention bias had an indirect effect in the association between BDD symptoms and PEs, in both directions. Additionally, we found that attention bias acted as a moderator in the link between PEs and BDD symptoms, such that a high level of attention bias might serve as an exacerbating factor against either BDD symptoms as PEs increase.

The limited evidence available supports our findings in several ways. There is sufficient empirical support from studies performed in clinical samples for a comorbidity between schizophrenia and BDD (Madeira et al. 2016; Stanghellini et al. 2012). A bidirectional link between psychotic symptoms and BDD is also supported by many previous studies. The presence of BDD was related to increased psychotic symptoms (Röhricht et al. 2020). Symptoms and signs of BDD were found to precede and exacerbate the development of a psychotic disorder (Abbassi et al. 2018; Durak et al. 2019; Lucchelli et al. 2006), and were suggested as ‘a marker of early schizophrenia’ (Stanghellini et al. 2012). Marshall et al. (2020) indicated that physical appearance‐related concerns fed into the content of voices, and represented a potential root of paranoia. On the other hand, the presence of paranoid thoughts was found to lead to more severe self‐reported body dysmorphic concerns (Toh et al. 2023). Recently, Rossell et al. (2020) observed that schizophrenia and BDD have overlapping symptoms, with BDD patients presenting with delusions with themes other than somatic (appearance‐related), and endorsing distress by persecutory and referential delusions to the same extent as schizophrenia patients. Beyond high comorbidity rates and some symptomatology overlap, multiple similarities can be noted between BDD and psychosis, including onset in adolescence or early adulthood, poor insight and significant impairment in academic, occupational or interpersonal functioning (Phillips et al. 2008). Overall, theoretical arguments may be presented for both directions, but empirical evidence is sparse. Due to a lack of sufficient knowledge regarding causal relationships between body dysmorphia and psychotic symptoms, and the pioneering nature of the present hypothetically driven model, further longitudinal studies are necessary to elucidate the predictive direction of the BDD‐PEs relationship.

Furthermore, mediation and moderation findings are in agreement with previous research and theory indicating that attention bias may be a prominent factor in the aetiology and early stages of body dysmorphia (Johnson et al. 2018) and psychosis (Johnson et al. 2018). Attention bias is suggested as one of the early predictors of the development of both BDD symptoms and positive psychotic symptoms. In light of our findings, and given the previous evidence showing that attentional bias and psychopathology tend to reinforce each other in a virtuous cycle (e.g., Moody et al. 2017), it can be suggested that when an individual attends to and processes a situation in a biassed manner, this impairs his/her experience of the situation, which bears more evidence for the attention bias and increases its accessibility and occurrence in the future. In other words, biassed attention towards potentially threatening stimuli may contribute to positive psychotic symptoms, which may induce body dysmorphic concerns, which can lead to a tendency to preferentially and rapidly attend to dysphoric stimuli, which may result in an increased proneness to PEs, and so on. These new findings enrich the literature in this area, and convey important clinical implications for guiding prevention and early intervention strategies.

## 5 Study Limitations

This study also has some limitations to be recognised and addressed in future research. The cross‐sectional design prevents any conclusions on the temporal order of symptoms or cause‐effect relations. The data were obtained from a community adolescent sample using a web‐based questionnaire and snowball sampling, which limits the representativeness and generalizability of findings. Future studies adopting probability sampling techniques and enlarging the sample size are needed. Study variables were assessed using a subjective self‐report measure, which may have led to response bias and social desirability bias. Further studies using semi‐structured interviews to assess PEs (such as the CAARMS) (Yung et al. 2002), and cognitive tasks (such as the Inverted Face Task) (Thompson 1980) or functional magnetic resonance imaging to assess attentional bias are still needed to confirm our findings.

## 6 Implications and Future Perspectives

Findings showed that attention bias had an indirect effect in the association between BDD symptoms and PEs in both directions, but moderated the association between PEs and BDD only (and not the reverse association BDD–PEs). One possible explanation is that PEs may represent broader vulnerability factors that sensitise adolescents to external threats, with attention biases amplifying these vulnerabilities and translating them into heightened body‐related concerns. By contrast, BDD does not seem to interact with attention bias strongly enough to exacerbate psychotic symptoms directly. In the real world, this suggests that attention bias may function as a non‐specific amplifier of psychotic vulnerability, but not as a universal amplifier of all pathways linking body image and psychosis. Although it is currently difficult to draw firm conclusions concerning the direction of the pathways between BDD and PEs before longitudinal studies are undertaken, this study's findings preliminarily suggest that the relationship linking these constructs seems to be complex and bidirectional. In light of these observations, it is crucial for clinicians to carefully evaluate psychotic symptoms in young people presenting with BDD symptoms, and vice versa, to assess whether those who endorse PEs also experience excessive appearance‐related concerns.

The present study will hopefully help to attract more research attention to this topic in the future at the national and international levels, and help to inform and galvanise efforts towards further understanding the connection between BDD and PEs, and achieving effective prevention and early intervention that can mitigate both conditions. In particular, more research is needed using clinical and non‐clinical samples to determine the relevance of each condition within the context of the other, and identify potential treatment targets for those at risk who endorse clinically significant PEs and dysmorphic concerns.

Intervention and prevention strategies targeting attention bias in young people with primary BDD presentations and who are at risk for psychosis may be promising clinical avenues and research directions. Specifically, implementing techniques of Cognitive Bias Modification (CBM) (Kuckertz and Amir 2017) could be used to help alleviate the effects of one condition on the other. CBM techniques aim at encouraging the individual to resolve ambiguous and threatening information (here, appearance‐related concerns) in a more positive way. This may be particularly relevant given the evidence supporting the effectiveness of CBM in mitigating symptoms of BDD (Premo et al. 2016; Summers and Cougle 2016) and psychosis (Yiend et al. 2023). In addition, the CBM treatment programme was previously tested online (e.g., Summers and Cougle 2016), which is highly practical for use with young people, especially those reluctant to seek care for mental health problems. The utility of targeting attentional bias in individuals with co‐existing BDD and PEs warrants further investigation. Results from these studies could then be used to inform the development of models to explain how BDD and PEs are directly and indirectly connected to each other through underlying cognitive mechanisms.

## 7 Conclusion

Yet the link of BDC to PEs at the mildest end of the psychosis continuum remains unexplored. The present study is an exploratory investigation of the bidirectional link between BDC and PEs in young people from the general population. Our results showed that attention bias acted as a moderator and a mediator in the relationship between BDD symptoms and PEs and in both directions. Of note, the indirect effect of attention bias was bidirectional, whereas the interaction effect was only unidirectional (from PEs to BDD, but not in the other direction). Keeping the study's limitations in mind, it may be concluded that attention bias could be a promising prevention and intervention target in individuals with PEs who also experience BDD symptoms. Future longitudinal research is required to examine the causal pathways between PEs and BDD symptoms and the role of attentional bias in these pathways, in order to offer more theoretical clarity.

## Ethics Statement

The research protocol underwent a thorough evaluation and received approval from the Ethics and Research Committee of the Notre Dame des Secours University Hospital. All participants provided their written informed consent before taking part and retained the option to withdraw from the survey at any point before submission. Informed consent to participate was obtained from the parents or legal guardians of any participant under the age of 16 years. All methods were done following the Declaration of Helsinki.

## Consent

The authors have nothing to report.

## Conflicts of Interest

The authors declare no conflicts of interest.

## Data Availability

The data that support the findings of this study are available on request from the corresponding author. The data are not publicly available due to privacy or ethical restrictions.
